# Comprehensive Analysis of the Potential Immune-Related Biomarker ATG101 that Regulates Apoptosis of Cholangiocarcinoma Cells After Photodynamic Therapy

**DOI:** 10.3389/fphar.2022.857774

**Published:** 2022-05-03

**Authors:** Zi-Jian Zhang, Kun-Peng Wang, Yun-Peng Huang, Chong Jin, Hao Jiang, Li Xiong, Zhao-Yi Chen, Yu Wen, Zhong-Tao Liu, Jing-Gang Mo

**Affiliations:** ^1^ Department of General Surgery, The Second Xiangya Hospital of Central South University, Changsha, China; ^2^ Department of General Surgery, Taizhou Central Hospital (Taizhou University, Hospital), Taizhou, China

**Keywords:** pan-cancer, cholangiocarcinoma, autophagy-related gene 101, apoptosis, photodynamic therapy, immune checkpoint gene

## Abstract

Autophagy related gene 101 (ATG101) plays a significant role in the occurrence and development of tumours by responding to stress. Our research aims to illustrate the correlation between the expression of ATG101 and tumor prognosis and its potential role and mechanism in tumor immunity and photodynamic therapy (PDT). First, integrated analysis of The Cancer Genome Atlas and Genotype-Tissue Expression portals were used to analyse the expression of ATG101. Then, Kaplan–Meier curves was applied in cholangiocarcinoma (CHOL) and liver hepatocellular carcinoma (LIHC) datasets for survival analysis. Next, the relationship between ATG101 expression and six immune cells, the immune microenvironment and immune checkpoints was analysed. Besides, the relationship between the expression of ATG101 and methyltransferase. GSEA was used to study the function and the related transcript factors of ATG101 in CHOL and LIHC. The effect of PDT on ATG101 was verified by microarray, qPCR and western blot. Then the effect of ATG101 and its regulatory factors on apoptosis were verified by siRNA, lentivirus transfection and Chip-qPCR. Comprehensive analysis showed that ATG101 was overexpressed in different tumours. Kaplan–Meier curves found that ATG101 was associated with poor prognosis in tumours (including CHOL and LIHC). We found that ATG101 can be used as a target and prognostic marker of tumour immunotherapy for different tumours. We also found that ATG101 regulates DNA methylation. GSEA analysis showed that ATG101 may play a critical role in CHOL and LIHC. Subsequent validation tests confirmed that the up-regulated ATG101 after PDT treatment is not conducive to the occurrence of apoptosis of cholangiocarcinoma cells. The high expression of ATG101 may be induced by the early stress gene EGR2. Our study highlights the significance of ATG101 in the study of tumour immunity and photodynamic therapy from a pan-cancer perspective.

## Introduction

Currently, cancer remains a serious health burden; in 2020, there are estimated to be more than 19.3 million new cases and nearly 10 million deaths from 36 cancers in 185 countries (Sung et al., 2021), placing a heavy burden on countries around the world (Ouyang et al., 2021). There is increasing evidence that immunotherapy and photodynamic therapy are potential treatments for various cancers (de Gooijer et al., 2020; O'Donnell et al., 2019). However, the immunological agents or photodynamic therapy (PDT) that have been used in the clinic cannot benefit all tumor patients, so more new targets related to immune or photodynamic therapy need to be explored. Autophagy related gene 101 (ATG101) is not only a key factor in autophagy, but also closely related to immune and stress responses in tumors, which can affect the occurrence and development of various tumors (Du et al., 2017). More importantly, in our unpublished microarray data, we found that PDT can induce the differential expression of ATG101 in gastric cancer, colorectal cancer and cholangiocarcinoma, so ATG101 may also be a common target for tumor immunity and photodynamic therapy. However, there are no studies on the significance of the association between ATG101, photodynamic therapy, and the cancer immune microenvironment.

Here, our study aimed to elucidate the correlation of ATG101 expression with tumor prognosis and its potential roles and mechanisms in tumor immunity and photodynamic therapy (PDT). We first analyzed the expression of ATG101 and its effect on survival by using data from TCGA and GTEx. In addition, we analyzed the relationship between ATG101 and immune cells, common immune checkpoint genes, and microenvironment scores, as well as the relationship between ATG101 expression levels and methyltransferase expression in various cancers. The expression of ATG101 was further verified by cholangiocarcinoma (CHOL) cell lines. And the effect of ATG101 on apoptosis of CHOL cells and its possible upstream regulatory mechanism were revealed. Our findings provide evidence for ATG101 as an immunotherapy target from a pan-cancer perspective and suggest that ATG101 can be used as a resistance marker for photodynamic therapy.

## Materials and Methods

### Obtain ATG101 Expression Data From Public Databases

The expression of ATG101 in various normal tissues and cell lines was analyzed using data from the GTEx and CCLE databases. Data from a total of 6678 normal tissues and organs were obtained from the GTEx database (https://www.gtexportal.org/home/). We collected 11057 samples from the TCGA database, including 10327 tumour samples and 730 paraneoplastic samples matched with 33 types of malignant cancers. To calculate whether there was a difference in ATG101 expression in tumors and non-tumors, some tumor samples with missing matches were first excluded. For the screened samples, we normalized the expression values of tumor samples and then performed the Kruskal-Wallis test to compare the differences in ATG101 expression between different tumors or cells. In addition, Wilcoxon test was used to compare the expression of ATG101 in tumor tissues and corresponding normal tissues. *p* < 0.05 was considered statistically significant.

### Correlation Between the Expression of ATG101 and the Prognostic Value in Various Cancer Patients

To verify whether the expression of ATG101 significantly affects the prognosis of tumours, the survival information (including the OS, DSS, DFS and PFS rates) of the patients from whom the 10327 tumour samples from 33 cancer types in the TCGA database were obtained was downloaded. The Kaplan-Meier survival estimation method was used to calculate the prognostic value of ATG101, and the log-rank test was used to determine the significant difference index. With *p* < 0.05 indicating a significant difference, the results obtained by these two methods were analyzed.

### Correlation Analysis Between Immune Cell Infiltration and ATG101 Expression

Through the TIMER algorithm, we used the gene expression profiles of 10897 samples from 32 cancer types in the TCGA database to infer the number of tumour-infiltrating immune cells (TIICs). TIMER is a statistical method of deconvolution that can be used for approximate calculation of immune infiltration. We analyzed the relationship between the expression level of the ATG101 gene and the abundance of infiltrating immune cells (including CD4^+^ T cells, CD8^+^ T cells, B cells, neutrophils, dendritic cells, and macrophages) through the above methods.

### Correlation Analysis Between the Expression Level of ATG101 and the Tumour Microenvironment

The non-tumour component cells in the tumour microenvironment, including immune cells and stromal cells, are mainly used for cancer diagnosis and prognostic evaluation. ESTIMATE (https://bioinformatics.mdanderson.org/public-software/estimate/) is a commonly used tool to predict the infiltration of stromal cells and immune cells in the tumour microenvironment and the purity of cancer tissues. According to the expression data from 9664 samples of 33 malignant cancer types, after excluding the normal tissue data, the ratio of stromal cells and immune cells in each tumour sample was calculated, and then the immune score, stromal score and ESTIMATE score were used to evaluate tumour purity. Furthermore, the ATG101 expression data and the score of the estimation algorithm were combined, and the Spearman correlation test was used to verify their correlation. This process mainly involved the following packages: ggplot2 (https://CRAN.R-project.org/package=ggplot2), ggpubr and ggExtra (https://CRAN.R-project.org/package=ggExtra).

### The Correlation of TMB, MSI and Neoantigens With the Expression of ATG101

Tumour mutation burden (TMB) and microsatellite instability (MSI) are important evaluation indicators directly related to the effect of immunotherapy, such as PD-1 blockade. The mutation data from 10114 samples from 33 cancer types were used to analyze the correlation between TMB and ATG101 expression levels. First, we calculated the mutation score of each tumour sample and obtained the TMB information for each cancer. In addition, we used the Spearman correlation test to analyze the correlation between ATG101 expression and TMB and used the fmsb software package (https://CRAN.R-project.org/package=fmsb) to construct a related radar chart. We also downloaded and analyzed the MSI scores of 10,415 tumour samples and used the same processing method to draw related radar charts of ATG101 expression and MSI. Predicted neoantigens of each sample were retrieved from a previous publication (Wen et al., 2019). The correlations between TMB, MSI, neoantigens and ATG101 expression were all calculated using the Spearman correlation test.

### Correlation of ATG101 With Immune Checkpoint Genes and Methyltransferase

Considering that the analysis results are intended to guide clinical practice, after exploring the relationship between ATG101 expressoin and immune cell infiltration, the correlation between ATG101 expression and various (47) immune checkpoint target genes was further analyzed. Here, the Spearman correlation test was used. Next, the reshape2 software package (http://www.jstatsoft.org/v21/i12/) was used to establish a related heat map. Changes in the DNA methylation state are common in tumours. DNA methylation plays an important role in the occurrence and development of tumours. Therefore, we deeply analyzed the correlation between four methyltransferases (DNMT1, DNMT2, DNMT3A and DNMT3B) and the expression level of ATG101. The analysis method was the same as that described above.

### Gene Set Enrichment Analysis Analysis

ATG101 expression is closely related to patient prognosis and immune regulation responsiveness in CHOL and LIHC; thus, we aimed to determine the potential mechanisms and signalling pathways of ATG101 in different tumours. First, according to the expression level of ATG101, the tumours were divided into high expression groups and low expression groups. Then, limma, org.Hs.eg.db and clusterProfiler (http://bioconductor.org/packages/release/bioc/html/clusterProfiler.html) were used to perform gene set enrichment analysis on tumours and to select the transcription factor information related to ATG101. Twenty-one common genes were obtained. These 21 genes were then analyzed by STRING (https://www.string-db.org/)) and visualised with Cytoscape.

### Quantification of Mutation/Neoantigens Load

Neoantigens are abnormally mutated proteins that are translated by cells and are recognized by antigen-presenting cells. Therefore, neoantigens have special significance in tumor diagnosis and treatment. We obtained variant effect predictor (VEP)-annotated somatic mutation data based on tumor neoantigens as defined by Rooney et al. (Wen et al., 2019). We used the NetMHCpan (v2.4) algorithm to predict whether cells in 33 tumors would produce tumor neoantigens. This algorithm can predict the affinity between peptides and major histocompatibility complex (MHC) class I molecules to define tumor neoantigens.

### Immunohistochemistry and KM Analysis

We obtained 31 human CHOL specimens and 86 LIHC specimens who underwent surgery at the investigator’s hospital from 2015 to 2017 and agreed to participate in the study. This experiment was approved by the Human Ethics Committee of the Second Xiangya Hospital of Central South University. After 5 years of follow-up by telephone or questionnaire, we found that the average and longest survival time of CHOL patients was 43 and 89 months, respectively. The average and longest survival times of LIHC patients were 57 and 85 months, respectively. Postoperative tissues from follow-up patients were embedded in paraffin and sections were prepared. Antibody incubation and diaminobenzidine (DAB) staining were performed following the instructions of a non-biotin detection kit (ZSGB-BIO, PV-9001, China). The stained sections were scored by pathologists, with high scores as the high expression group and low scores as the low expression group.

### Cell Lines

RBE and HUCCT1 were purchased from the Cell Resource Centre of Shanghai Institutes for Biological Sciences and maintained in RPMI-1640 medium supplemented with 10% foetal bovine serum. Cell lines were routinely cultured at 37°C, with 21% O_2_ and 5% CO_2_.

### Annexin-V-FITC/PI Assay

Annexin-V-FITC/PI assay was performed with indicated antibodies using Annexin-V-FITC/PI Kits (Yeasen Technology, 40302ES20, China) according to manufacture instructions. Briefly, CHOL cells were treated with different photodynamic therapies, trypsinized without EDTA and resuspended in binding buffer, and then stained with Annexin-V-FITC followed by PI for 10 and 15 min in the dark, respectively. Cells were analyzed for apoptosis by using a flow cytometer (Beckman Coulter Epics Altra, Miami, FL).

### RNA Extraction and Quantitative Real-Time PCR

Total RNA was extracted by sequentially adding TRIzol reagent (Invitrogen, USA), chloroform, isopropanol and ethanol. cDNA was obtained by reverse transcription of 1 μg of total RNA using PrimeScript RT kit (Perfect Real-Time, TaKaRa). RNA expression levels are measured by amplifying the gene of interest in cDNA using a quantitative real-time PCR system. The primers were provided by the Shenggong Company (Supplementary Table S1).

### Western Blotting

Protein samples of CHOL cells were lysed using radioimmunoprecipitation assay (RIPA) buffer. Add sodium dodecyl sulfate (SDS) loading buffer to the samples and heat at 95°C for 5 min. Electrophoresis was performed at 120 V, 50 min, and protein transfer was performed using a polyvinylidene fluoride (PVDF) membrane at 400 mA, 45 min. After PVDF membrane blocking, the following primary antibodies ATG101 (1:1000, AB229235, Abcam), ATF4 (1:1000, #11815, CST), PERK (1:1000, #3192, CST), EGR2 (1:1000, #13491-1-AP, Proteintech) and β-actin (1:1000, #20536-1-AP, Proteintech) were added overnight. Primary antibodies were labeled with a horseradish peroxide (HRP)-conjugated secondary antibody and visualized using an electrochemiluminescence (ECL) luminescence solution. The results were photographed and stored by a multifunctional imager (Amersham Imager 600, GE, American).

### Small Interfering RNA and Lentivirus Transfection

We ordered 2 small interfering RNAs (siRNAs) to knock down ATG101 mRNA in the CHOL cell line (RiboBio, Guangzhou, China). 10 × 10^4^ cells were seeded into 6-well plates and transfected with siRNA using lipo8000 (Biyuntian, Shanghai, China) after reaching 30% confluency. After 48 h of incubation, the efficiency of knockdown was assessed using qRT-PCR or Western blot. The target sequences of the ATG101 siRNAs in ATG101 were as follows: siATG101-1, 5′-ACU​UCA​UCG​ACU​UCA​CUU​ATT-3′; siATG101-2, 5′-CAG​CCC​UAC​CUG​UAC​AA-3′; siNC, 5′-UUC​UUC​GAA​GGU​GUC​ACG​UTT-3′.

### ChIP-qPCR Assay

ChIP was performed with indicated antibodies using ChIP Assay Kits (Beyotime, P2078, China) according to manufacture instructions. Primer sequences used for the amplification of human ATG101 promoter associated with EGR2 were 5′- CTG​GTC​GTG​GAC​TGT​GGT​TG-3′ (forward) and 5′- CGGAAGCTGGAGGAGCG -3′ (reverse). Control Primer was supplied by Kit.

### Cell Proliferation Assays and Colony Formation Assays

HUCCT-1 and RBE cells were seeded in 6-well or 96-well plates after 24 h, the liquid in the plates was changed to medium containing 0–300 nM pyropheophorbide-alpha (PYRO). After 4 h of incubation, the plates were irradiated with a 630 nm wavelength laser so that the total energy density absorbed by the cells reached 10 J/cm2. In transfection assays, we seeded RBE and HUCCT1 in 6-well plates and transfected with siATG101-1, siATG101-2 or siNC for 48 h at 37°C with 5% CO_2_. Then 10 μl Cell Counting Kit-8 solution (CCK-8, #C0037, Beyotime, Shanghai, China) was added and incubated for 20 min. The absorbance of formazan was detected at 450 nm under microplate reader (Thermo Scientific, CA, United States).

### Subcutaneous Xenograft Model

All animal experiments were approved by the Scientific Investigation Board of the Central South University (Changsha, China) and performed in accordance to the National Institute of Health Guide for the Care and Use of Laboratory Animals. All female BALB/c nude mice (4–8 weeks old) were purchased from SJA Laboratory Animals (Changsha, China) and housed in a specific pathogen-free facility. Nude mice were subcutaneously injected with 5 × 10^6^ CHOL cells, and treatment was started when the subcutaneous tumor volume reached to 50 mm^3^. After randomization, 0.1 ml of PYRO or PBS (*n* = 3/group) was administered by intraperitoneal injection twice a week. The body weight of the mice was measured weekly. Mice were sacrificed 3 weeks after inoculation, and tumor tissues were isolated for weighing and Western blot assays.

### Statistical Analysis

All data in this study were analyzed using R version 4.0.3 (https://www.r-project.org/) and its auxiliary software packages, and all analyses were performed with *p* < 0.05 as the standard of significant difference. We used the limma package to determine the expression level of ATG101 in tumors and the Wilcoxon test to calculate the significant difference. KM analysis was used to analyze the prognostic value of ATG101 using the log-rank test. Spearman method was applied to analyze the correlations between ATG101 and immune cells and immune genes.

## Results

### Expression of ATG101 in Pan-Cancer

**FIGURE 1:**
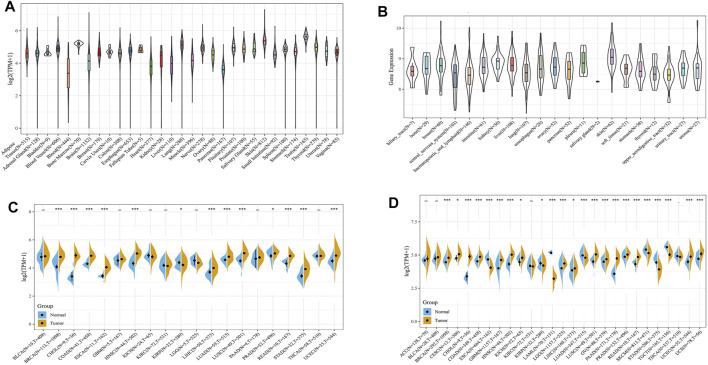
The expression level of ATG101 in different cancer patients. **(A)** The expression of ATG101 in 31 normal tissues from the GTEx database. **(B)** The expression of ATG101 in 21 types of malignant cancer cells from the CCLE database. **(C)** The expression of ATG101 between 20 types of malignant tumour tissues and normal tissues from the TCGA database. **(D)** The expression of ATG101 in 27 types of malignant tumour tissues from the TCGA database and the corresponding normal tissues from the GTEx database. *p < 0.05, **p < 0.01, ***p < 0.001.

First, we compared tumour samples and paracarcinoma tissues from the TCGA database, normal samples from the GTEx database and cancer cells from the CCLE database to evaluate the mRNA expression characteristics of ATG101 in humans. We found that the expression of ATG101 varied in different normal tissues (Figure 1A) and cancer cells (Figure 1B) after collating the tumour tissues and paracarcinoma tissues from the TCGA database. We found that the expression of ATG101 was upregulated in BRCA, CHOL, COAD, ESCA, HNSC, LIHC, LUAD, LUSC, PRAD, READ, STAD, and UCEC. ATG101 was downregulated only in KIRP (Figure 1C). Then, we analyzed the difference in ATG101 expression between normal samples from the GTEx database and tumour tissues from the TGGA database and found that ATG101 expression was upregulated in BRCA, CESC, CHOL, COAD, GBM, HNSC, KICH, LGG, LIHC, LUSC, OV, PAAD, PRAD, READ, SKCM, UCEC and UCS. However, its expression was found to be downregulated in ESCA, LAML, LUAD, SKCM, STAD and TGCT (Figure 1D).

### Correlation Analysis Between ATG101 Expression Level and Prognostic Value

**FIGURE 2:**
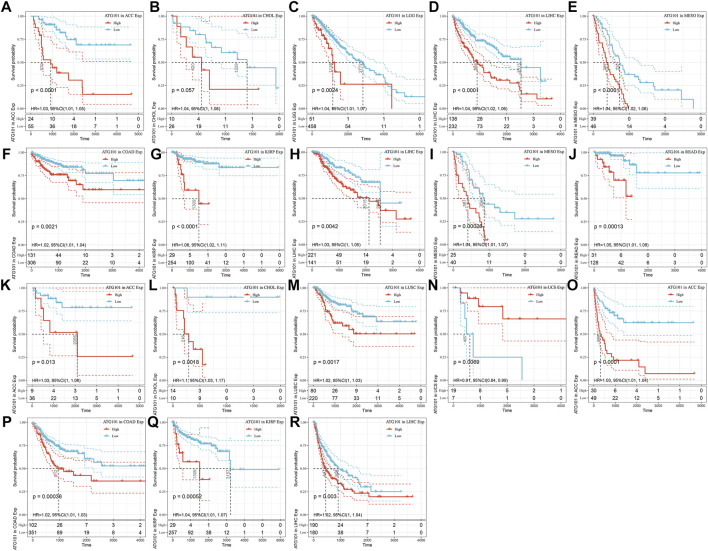
The relationship between the expression of ATG101 and the prognosis of patients with 33 types of malignant tumours from the TCGA database. **(A–E)** Overall survival. **(F–J)** Disease-specific survival. **(K–N)** Disease-free survival. **(O–R)** Progression-free survival.

The characteristics of ATG101 expression at the mRNA level suggested that ATG101 may be a valuable target for pan-cancer. Therefore, we further used Kaplan–Meier analysis to explore the correlation between ATG101 mRNA expression levels and the survival outcomes (including the OS, DSS, DFS and PFS rates) of different cancers from the TCGA database. Our results demonstrated that upregulated ATG101 expression was associated with a shorter OS rate in ACC, CHOL, LGG, LIHC, and MESO (Figures 2A–E). In addition, upregulation of ATG101 expression was related to a shorter DSS rate in ACC, COAD, KIRP, LGG, LIHC, MESO and READ (Figures 2F–L). High ATG101 expression was associated with a poor DFS rate in ACC, CHOL and LUSC, while low ATG101 expression was associated with a poor DFS rate in USC (Figures 2M–P). Furthermore, the high expression of ATG101 was also associated with a poor PFS rate in ACC, COAD, KIRP and LIHC (Figures 2Q–T). These results confirmed that the high expression of ATG101 is negatively correlated with the prognosis of most malignant tumours.

### Correlation Analysis Between ATG101 Expression and Immune Cells

**FIGURE 3:**
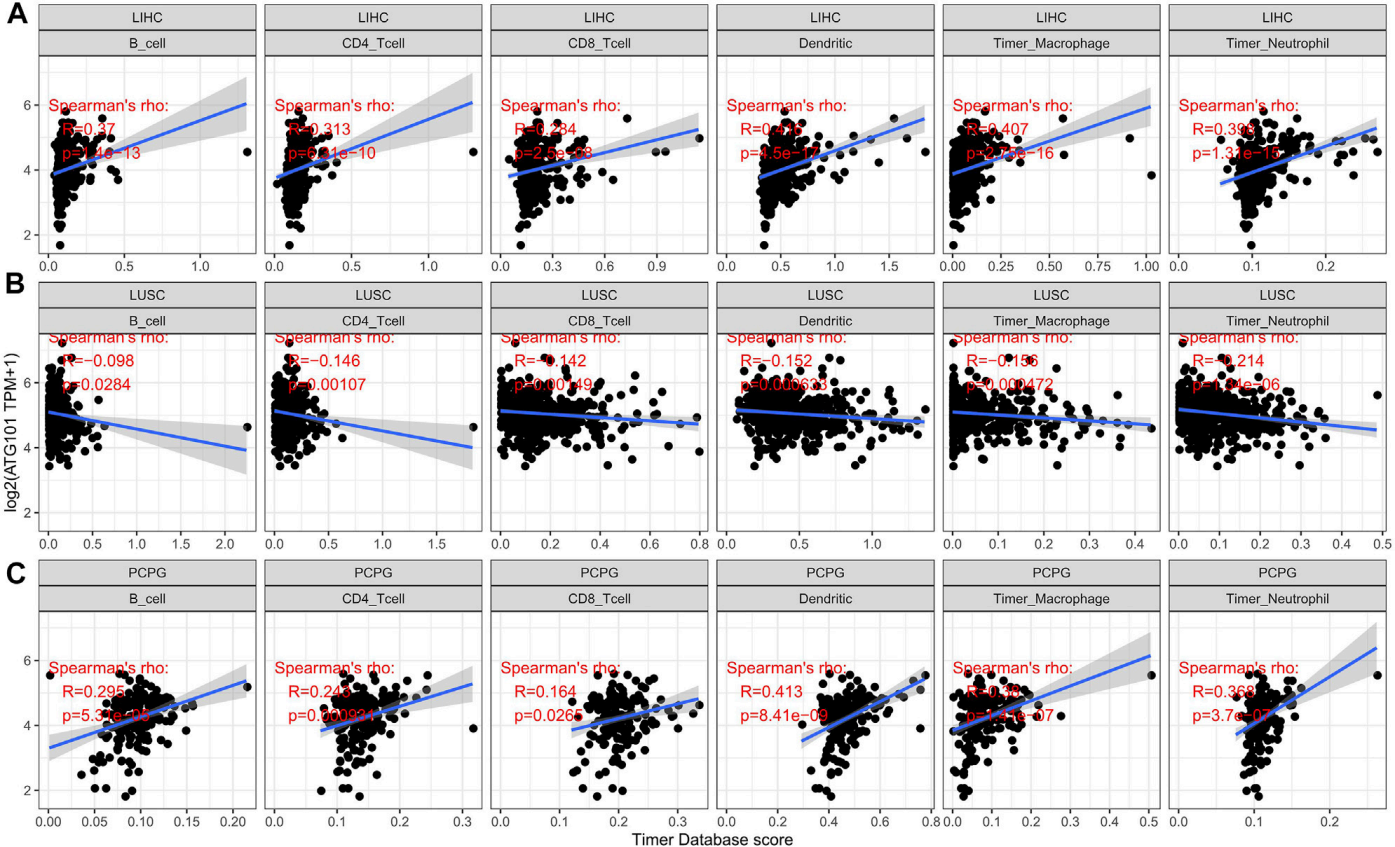
The relationship between the expression level of ATG101 and the infiltration level of six kinds of immune cells in patients with malignant tumours. **(A–C)** The three tumours, LIHC, LUSC and PCPG, respectively, with the strongest correlation with immune cell infiltration.

We explored the relationship between ATG101 expression and immune infiltration in different tumours after investigating the relationship between ATG101 expression and cancer prognosis. An analysis of six tumour-infiltrating immune cells (B cells, CD4^+^ T cells, CD8^+^ T cells, neutrophils, macrophages and dendritic cells) was conducted. With the cut-off of *p* value of 0.05, ATG101 expression was positively correlated with tumour-infiltrating immune cells. In LIHC (Figure 3A), ATG101 expression was positively correlated with B cells (correlation coefficient = 0.37, *p* value = 1.4e-13), CD4^+^ T cells (correlation coefficient = 0.313, *p* value = 6.31e-10), CD8^+^ T cells (correlation coefficient = 0.284, *p* value = 2.5e-08), dendritic cells (correlation coefficient = 0.416, *p* value = 4.5e-17), macrophages (correlation coefficient = 0.407, *p* value = 2.75e-16) and neutrophils (correlation coefficient = 0.398, *p* value = 1.31e-15). In LUSC (Figure 3B), ATG101 expression was positively correlated with B cells (correlation coefficient = 0.098, *p* value = 0.0284), CD4^+^ T cells (correlation coefficient = 0.146, *p* value = 0.00107), CD8^+^ T cells (correlation coefficient = 0.142, *p* value = 0.00149), dendritic cells (correlation coefficient = 0.152, *p* value = 0.000633), macrophages (correlation coefficient = 0.156, *p* value = 0.000472) and neutrophils (correlation coefficient = 0.214, *p* value = 1.34e-06). In PCPG (Figure 3C), ATG101 expression was positively correlated with B cells (correlation coefficient = 0.295, *p* value = 5.31e-09), CD4^+^ T cells (correlation coefficient = 0.243, *p* value = 0.000931), CD8^+^ T cells (correlation coefficient = 0.164, *p* value = 0.0265), dendritic cells (correlation coefficient = 0.413, *p* value = 8.41e-09), macrophages (correlation coefficient = 0.38, *p* value = 1.41e-07) and neutrophils (correlation coefficient = 0.368, *p* value = 3.7e-07).

### Relationship Between ATG101 Expression and the Tumour Immune Microenvironment

**FIGURE 4:**
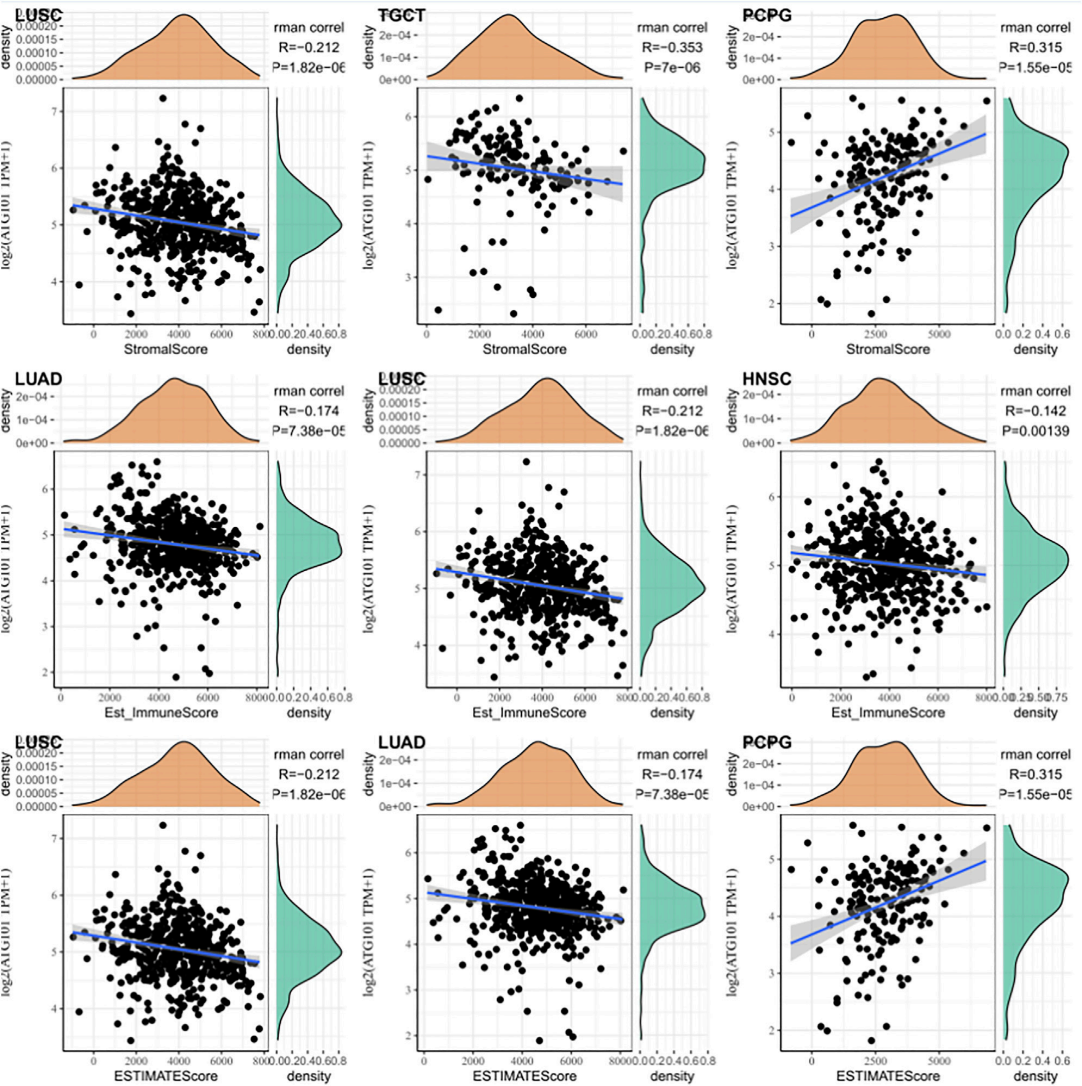
The relationship between the algorithm score in cancer patients and the expression level of ATG101. The third highest correlations with the stromal score, immune score and ESTIMATE score.

Based on the correlation between immune cells and ATG101 expression, we also explored the relationship between ATG101 expression and the tumour immune microenvironment. Our results showed that ATG101 expression was significantly positively correlated with the immune score in BLCA, BRCA, CESC, COAD, DLBC, ESCA, GBM, HNSC, KICH, KIRC, LAML, LGG, LIHC, LUAD, LUSC, PCPG, SARC and UVM (Supplementary Figure S1). ATG101 expression was significantly positively correlated with stromal score in BLCA, BRCA, CESC, COAD, GBM, HNSC, KICH, KIRP, LGG, LIHC, LUAD, LUSC, MESO, PCPG, PRAD, SARC, STAD, TGCT, THCA, THYM, UCEC and UVM (Supplementary Figure S2). Moreover, ATG101 expression was significantly positively correlated with the ESTIMATE score in BLCA, BRCA, CESC, COAD, ESCA, GBM, HNSC, KICH, LAML, LGG, LIHC, LUAD, LUSC, MESO, PCPG, SARC and UVM (Supplementary Figure S3). ATG101 expression was found to be significantly positively correlated with all three scores in BLCA, BRCA, CESC, COAD, GBM, HNSC, KICH, LGG, LIHC, LUAD, LUSC, PCPG, SARC and UVM. Overall, ATG101 expression had the third highest correlation with stromal score in LUSC (r = −0.212, *p* < 0.001), TGCT (r = −0.353, *p* < 0.001), and PCPG (r = 0.315, *p* < 0.001). ATG101 expression had the third highest correlation with immune score in LUAD (r = −0.174, *p* < 0.001), LUSC (r = -0.212, *p* < 0.001), HNSC (r = −0.142, *p* < 0.001) and the third highest correlation with the ESTIMATE score in LUSC (r = −0.212, *p* < 0.001), LUAD (r = −0.174, *p* < 0.001), PCPG (r = 0.315, *p* < 0.001) (Figure 4).

### Relationship Between ATG101 Expression and Immune Related Gene in Various Cancers

**FIGURE 5:**
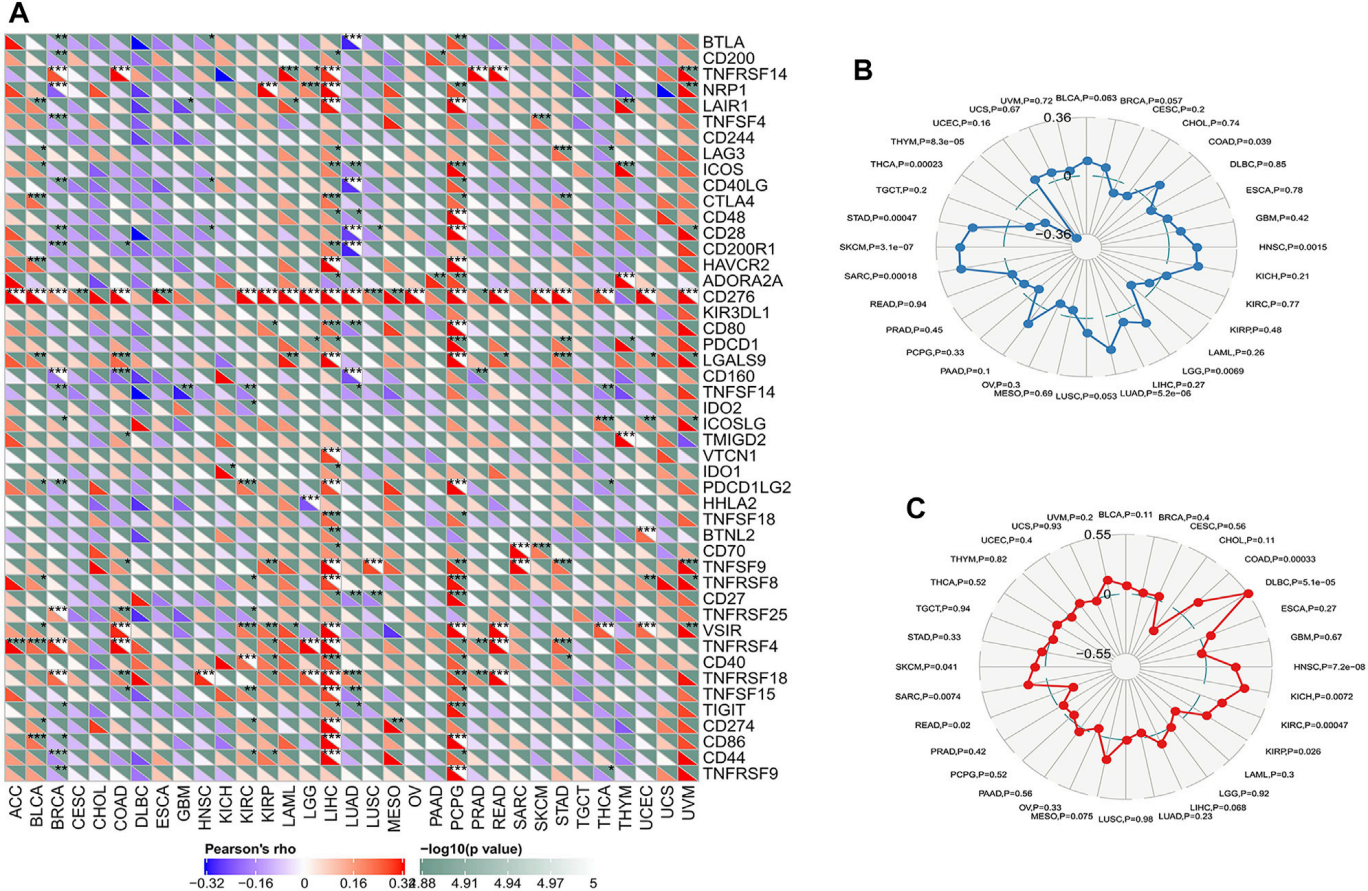
The relationship between ATG101 expression and immunoreactivity. **(A)** The correlation between immune checkpoint gene expression and ATG101 expression level. *p < 0.05, **p < 0.01, ***p < 0.001. **(B)** The relationship between ATG101 expression level and TMB. **(C)** ATG101 expression level and microsatellite instability (MSI) in tumour patients.

The immune checkpoint is an inhibitory signalling pathway in the immune system that regulates the intensity and persistence of the immune response in peripheral tissue, prevents tissue injury and plays an important role in maintaining self-antigen tolerance. We used the mRNA sequence database to assess whether there was an association between the expression level of ATG101 and 47 common immune checkpoint genes. We found that ATG101 was highly correlated with immune checkpoint genes in various cancers. In BRCA, COAD, LIHC, LUAD and PCPG, we found that ATG101 and other immune checkpoint genes were significantly coexpressed. However, the expression of ATG101 was negatively correlated with all forty-seven immune checkpoint genes in CHOL, DCBC, TGCT and UCS (Figure 5A).

Classic immune checkpoint therapeutic targets, including PD-1, PD-L1 and CTLA-4, are widely targeted clinically and these therapies have achieved satisfactory effects. TMB and MSI are important evaluation indexes directly related to the efficacy of immune checkpoint therapies such as PD-1 blockade. We found that TMB was significantly correlated with the expression levels of ATG101 in COAD, HNSC, LGG, LUAD, SARC, SKCM, STAD, THCA and THYM (*p* < 0.05). In addition, UCEC had the lowest TMB score, and SKCM had the highest. (Figure 5B). The results showed that ATG101 expression is negatively correlated with the hypermutation state in UCE but positively correlated with the hypermutation state in SKCM. We also researched the correlation between ATG101 expression and MSI in various cancers. We found that ATG101 expression was significantly correlated with MSI in COAD, DLBC, HNSC, KICH, KIRC, KIRP, READ, SARC and SKCM (*p* < 0.05), while DLBC had the highest coefficient and CHOL had the lowest score (Figure 5C).

### Relationship Between ATG101 Expression and the Expression of Four Methyltransferases in Various Cancers

**FIGURE 6:**
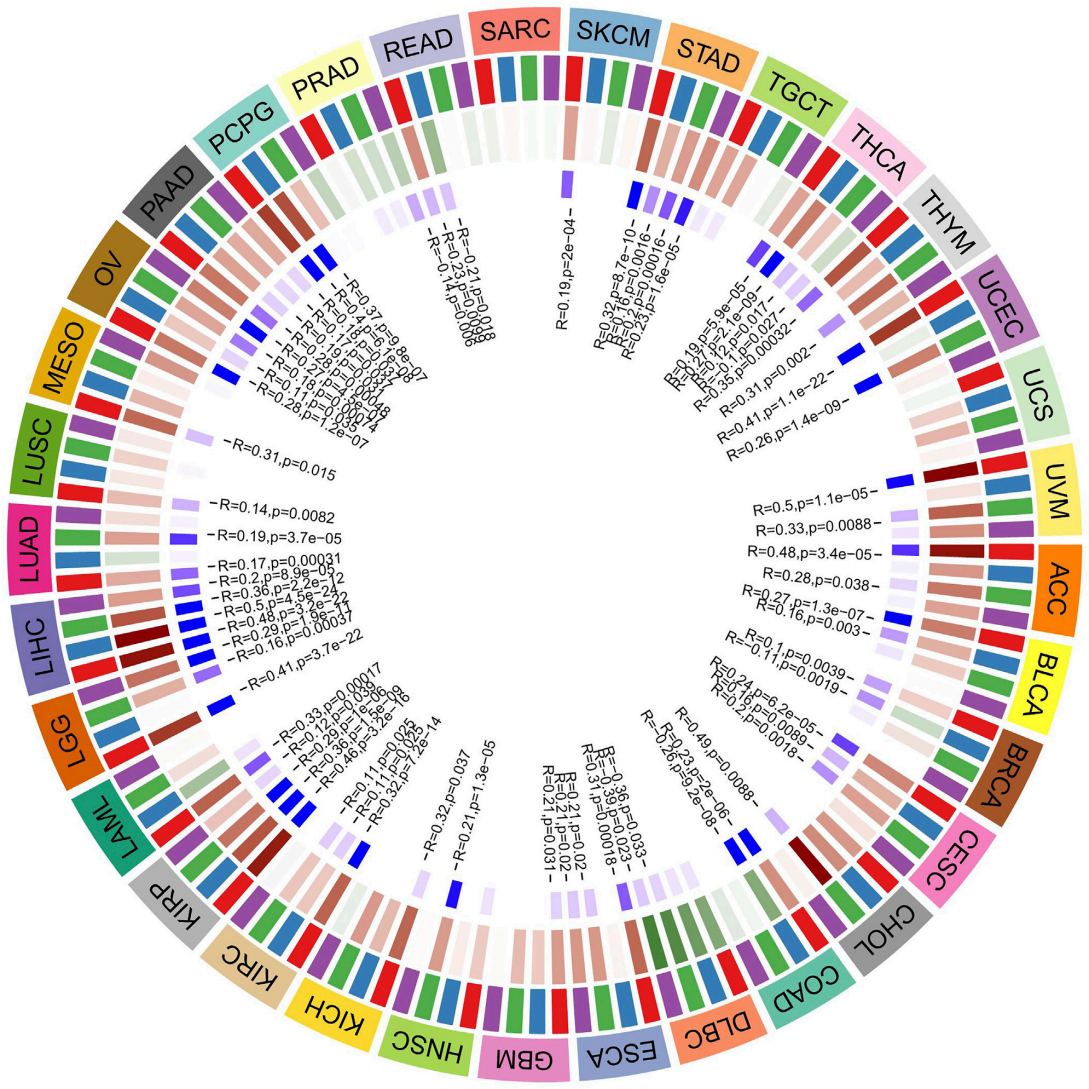
The relationship between 4 methyltransferases and the expression level of ATG101. The darker the blue square in the inner circle represents the smaller the *p* value of the corresponding tumor. The red square in the middle circle represents a positive correlation, and the green represents a negative correlation, and the darker the color, the higher the correlation.

DNA methylation is a physiological process that changes chromatin structure, DNA conformation, DNA stability, and the interaction between DNA and protein through the action of DNA methyltransferase. It is one of the main mechanisms by which gene expression is regulated. DNA methylation is considered to be one of the main factors affecting tumour occurrence and development. In our research, we explored the correlation between ATG101 expression and the expression of four methyltransferases (DNMT1, DNMT2, DNMT3A and DNMT3B) (Figure 6). In STAD, THCA, THYM, UCEC, UVM, ACC, BLCA, BRCA, CESC, CHOL, COAD, DLBC, ESCA, GBM, HNSC, KICH, KIRC, KIRP, LGG, LIHC, LUAD, LUSC, MESO, OV, PAAD, PCPG, PEAD and SKCM, ATG101 expression was positively correlated with DNA methylation (*p* < 0.05). In contrast, ATG101 expression was negatively correlated with DNA methylation in UCS, SARC, and TGCT (*p* < 0.05).

### Survival Analysis and Gene Set Enrichment Analysis of ATG101 in CHOL and LIHC

**FIGURE 7:**
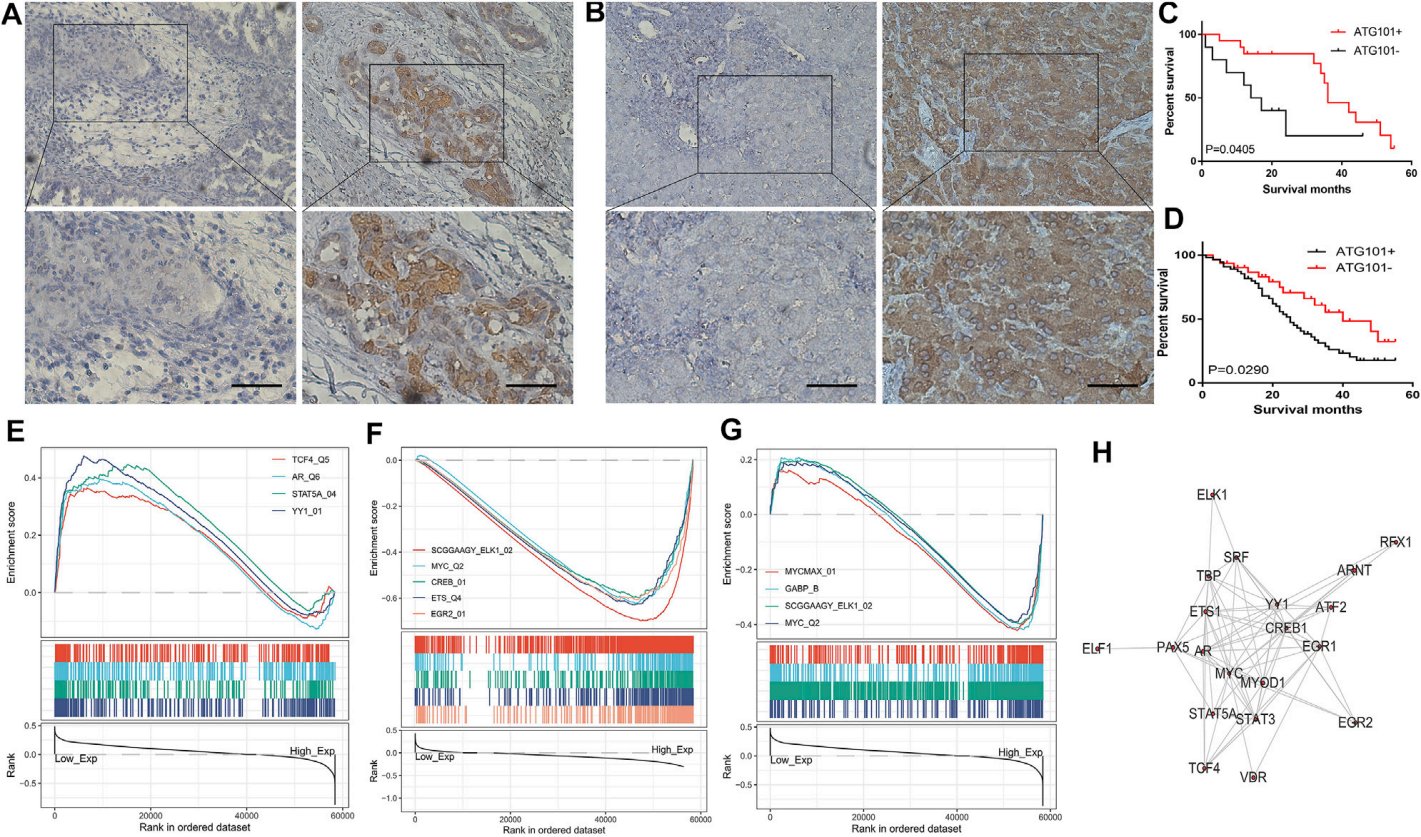
Immunohistochemical staining of CHOL and LIHC. The samples were obtained from patients after surgery in hospitals. **(A,B)** The survival analysis curve of CHOL and LIHC. **(C,D)** The pathways are negatively and positively correlated with ATG101 expression in LIHC. The transcription factors are positively and negatively correlated with ATG101 expression in CHOL **(E,F)**. The transcription factors are correlated with ATG101 expression in LIHC **(G)**. Twenty-one common genes related to ATG101, CHOL and LIHC **(H)**.

We analyzed the expression of ATG101 in clinical samples obtained from CHOL and LIHC patients. We collected 31 tissue samples of CHOL and 86 tissue samples of LIHC, and performed immunohistochemical staining to determine the expression of ATG101. Medium or strong staining was observed in 35.48% (11/31) of CHOL specimens, indicating ATG101 expression, while 64.52% (21/31) of samples showed weak staining, indicating negative ATG101 expression. Figure 7A shows representative images of CHOL samples with strong, medium, or weak ATG101 expression. In LIHC tissues, 63.95% (55/86) of the samples showed medium or strong staining, indicating positive ATG101 expression, and 36.05% (31/86) of the samples showed weak or negative staining, indicating negative ATG101 expression. A representative image of the LIHC sample is shown in Figure 7B. In addition, we divided CHOL and LIHC specimens into 2 groups according to the negative or positive expression of ATG101. The KM method was used to analyze the relationship between OS and ATG101 in patients with CHOL or LIHC; the results of this analysis showed that the OS of patients with positive ATG101 expression was lower than that of patients with negative expression in CHOL and LIHC (*p* = 0.0405, *p* = 0.0290, respectively) (Figures 7C,D).

After preliminary exploration of the genetic and epigenetic mechanisms of ATG101, we used GSEA to research the function of ATG101 in CHOL and LIHC. As for transcription factor level, ATG101 can promote the function of transcription factor 4, androgen receptor, signal transducer and activator of transcription 5A and YY1 transcription factor (Figure 7E). In addition, ATG101 can inhibit the function of members of the ETS oncogene family, v-myc avian myelocytomatosis viral oncogene homologue, cAMP responsive element binding protein 1, v-ets avian erythroblastosis virus E26 oncogene homologue and early growth response 2 (Figure 7F). In LIHC, ATG101 can affect the function of v-myc avian myelocytomatosis viral oncogene homologue, GA binding protein transcription factor alpha subunit, ETS oncogene family, v-myc avian myelocytomatosis viral oncogene homologue and v-myc avian myelocytomatosis viral oncogene homologue (Figure 7G). The transcription factor binding information for ATG101 was then downloaded from ChIPBase, and the genes enriched by GSEA were intersected with the genes identified from ChIPBase. Twenty-one common genes related to ATG101, CHOL and LIHC were obtained (Figure 7H).

### PYRO-PDT Induces Apoptosis and Up-Regulates the Expression of ATG101, ATF4 and PERK

**FIGURE 8:**
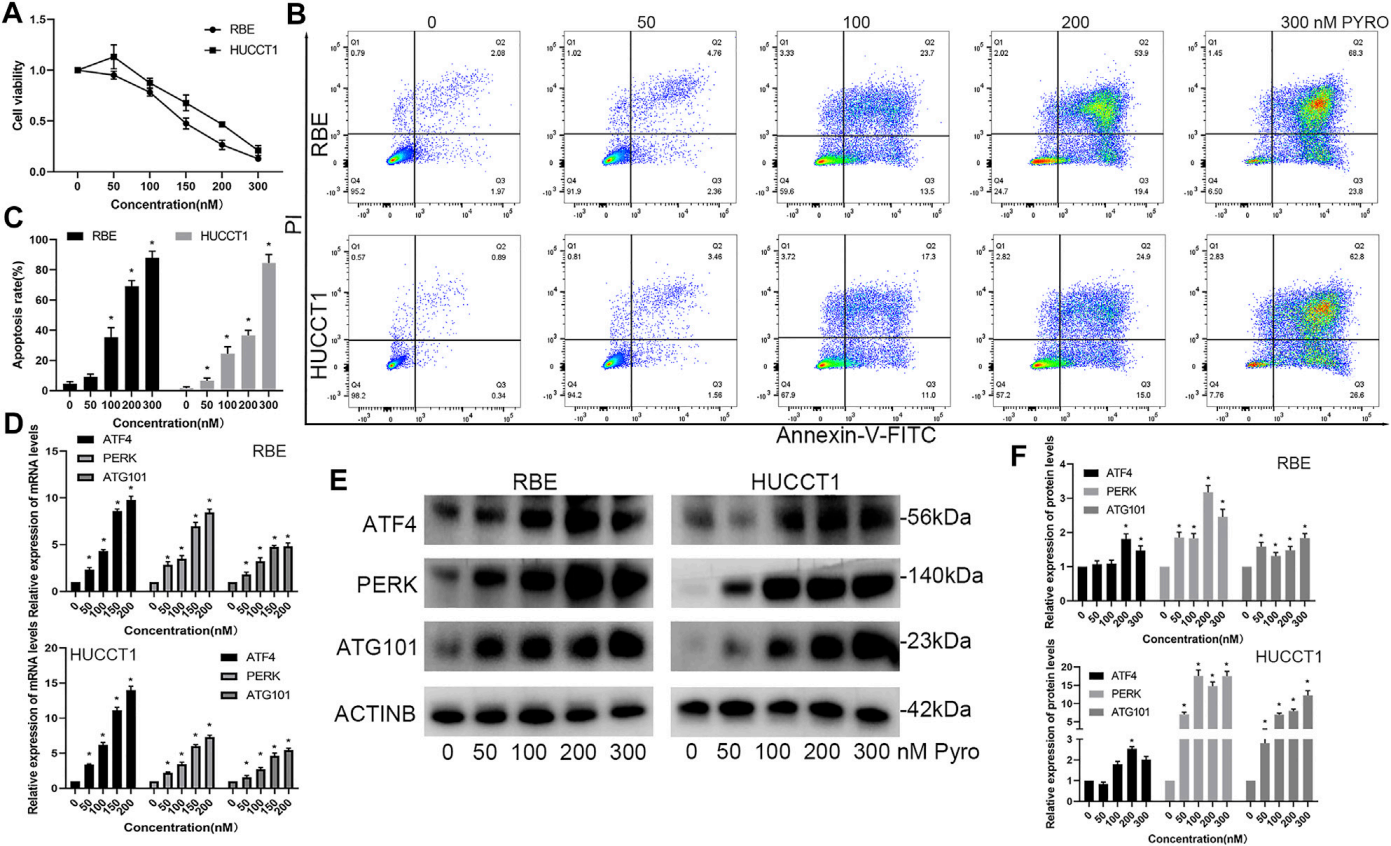
PYRO-PDT induces apoptosis and up-regulates the expression of ATG101 and unfolded protein response markers. **(A)** The effect of different concentrations of PYRO-PDT (0–300 nM, 10J/cm^2^) on the viability of RBE and HUCCT1 cells in a 96-well plate was detected by CCK8 assay. **(B)** After treating RBE and HUCCT1 cells in a 6-well plate with different concentrations of PYRO-PDT for 4 h, the apoptosis level was detected by Annexin-V-FITC/PI staining and flow cytometry. **(C)** is a histogram of the early and late apoptosis rates of different groups in **(B)**. **(D)** After processing the RBE and HUCCT1 cell lines in 6-well plates with different concentrations of PYRO-PDT for 4 h, the mRNA levels of ATF4, PERK and ATG101 were determined by qRT-PCT. **(E)** Determine the protein levels of ATF4, PERK and ATG101 by western blot after the same treatment as in **(D)**. **(F)** is a histogram of the relative expression levels of proteins in each group in **(E)**. *p < 0.05.

In our previous microarray data (Tan et al., 2019; Zheng et al., 2021), it was found that many tumors including CHOL and colorectal cancer, up-regulated ATG101 after PDT treatment. The current view believes that the main anti-tumor effect of PDT is to induce cell apoptosis. Therefore, we would like to further explore the effect of ATG101 in the apoptosis of CHOL cells after PDT treatment. First of all, we determined the concentration range of PYRO-PDT to effectively inhibit the viability of CHOL cells, and determined that PYRO can reach IC50 of RBE and HUCTT1 cells when the concentration is about 150 and 200 nM, respectively (Figure 8A). On this basis, we found that 0–300 nM PYRO-PDT can gradually increase the apoptosis rate of CHOL cells (Figures 8B,C). Our previous studies (Wen et al., 2019) have found that the unfolded protein response and endoplasmic reticulum stress are the most common cellular responses induced after PDT. In the TCGA-CHOL data, there is a significant correlation between ATG101 and the non-folded protein response markers ATF4 and PERK (Supplementary Figure S5). Therefore, we simultaneously detected the mRNA and protein of the above three molecules in RBE and HUCCT1 cells after PDT treatment. The results suggest that PDT can not only induce apoptosis, but also the three are significantly up-regulated and are dependent on the concentration of PYRO (Figures 8D–F).

### Down-Regulation of ATG101 Inhibits the ATF4, PERK and Promotes PDT-Induced Apoptosis Rate

**FIGURE 9:**
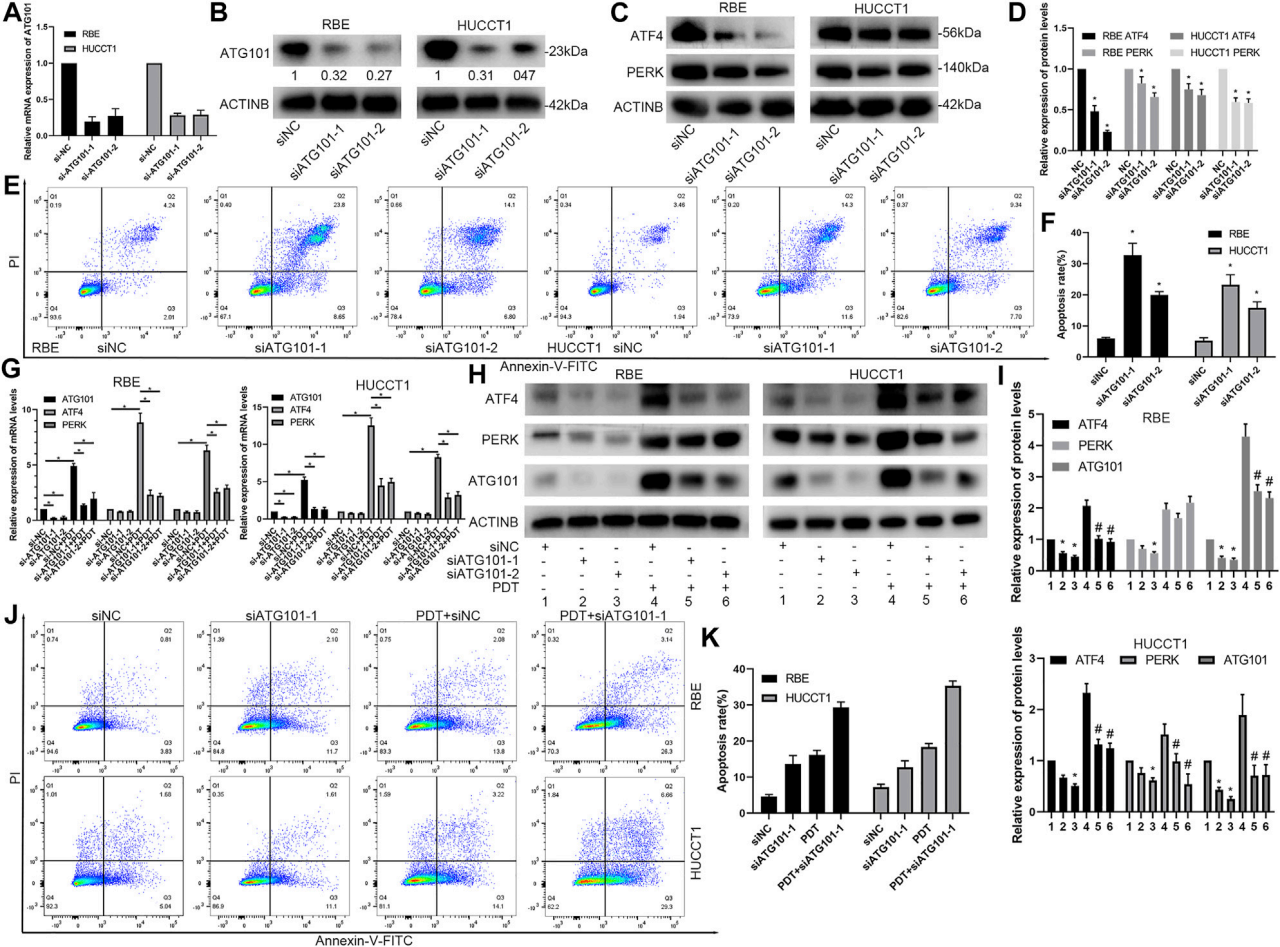
Downregulation of ATG101 inhibits the expression of unfolded protein response markers and promotes PDT-induced apoptosis of cholangiocarcinoma cells. **(A,B)** ATG101 was depleted by specific siRNA (siATG101-1, siATG101-2) in RBE and HUCCT1 cell lines and confirmed by qRT-PCR(A) or Western blot(B). **(C)** ATG101 was depleted by specific siRNA in RBE and HUCCT1 cell lines and protein levels of ATF4, PERK were confirmed by western blot. **(D)** is a histogram of the relative expression levels of proteins in each group in **(C)**. **(E)** Different siRNAs (siNC, siATG101-1, siATG101-2) were transfected with RBE and HUCCT1 cells in a 6-well plate for 48 h, and then the level of apoptosis was detected by Annexin-V-FITC/PI staining and flow cytometry. **(F)** is a histogram of the total apoptosis rate of each group in **(E)**. **(G,H)** Comparison of effect on ATF4, PERK and ATG101 by qRT-PCR and western blot in different siRNAs groups (siNC, siATG101-1, siATG101-2) which were transfected with RBE and HUCCT1 cell lines for 40 h with or without PYRO-PDT (100 Nm, 10 J/cm^2^). **(I)** is a histogram of the relative expression of proteins in each group in (H). * Compared with group 1; ^#^ compared with group 4. **(J)** Annexin-V-FITC/PI staining and flow cytometry was used to detect the different levels of ATG101 (siNC, siATG101-1) and whether combined with PDT affects apoptosis. **(K)** is a histogram of the total apoptosis rate of each group in **(J)**. *, ^#^ *p* < 0.05.

PDT can simultaneously induce apoptosis, high expression of ATG101, and unfolded protein reaction, but the relationship between the three has not been clarified. Therefore, we designed 2 different siRNA products (siATG101-1 and siATG101-2) to explore their effects on molecules such as PDT and ATF4. First, the results of a separate siRNA transfection test showed that siAT101 can effectively inhibit the mRNA and protein expression of ATG101 (Figures 9A,B). After down-regulating ATG101, ATF4 and PERK in RBE and HUCCT1 cells also decreased (Figures 9C,D). Then, we tested the effect of knocking down ATG101 alone on apoptosis using an apoptosis kit, and found that the group with low ATG101 expression in RBE and HUCCT1 cells also had a higher rate of apoptosis under non-stress conditions (Figures 9E,F). Furthermore, we tested the effect of ATG101 on apoptosis and unfolded protein markers under the stress level induced by PYRO-PDT. The results of qPCR and western blot showed that under the stress conditions caused by PDT, ATG101 was significantly up-regulated. Knockdown of ATG101 by siRNA can inhibit the mRNA and protein expression of ATF4 and PERK. In the absence of PDT, knockdown of ATG101 can also inhibit the mRNA or protein expression of ATF4 and PERK, but the difference is not as significant as under stress (Figures 9G–I). Finally, in the experiment of the effect of siATG and PDT on apoptosis, we found that knockdown of ATG101 alone can increase the apoptosis rate of CHOL cells, and more importantly, knockdown of ATG101 under the background of PDT, it can significantly increase the level of apoptosis (Figures 9J,K). Therefore, these results suggest that ATG101, which is upregulated by PDT, may have an antagonistic effect on apoptosis. Inhibiting ATG101 on the basis of PDT can increase the anti-tumor effect.

### EGR2 Regulates the Expression of ATG101 After PYRO-PDT Treatment

**FIGURE 10:**
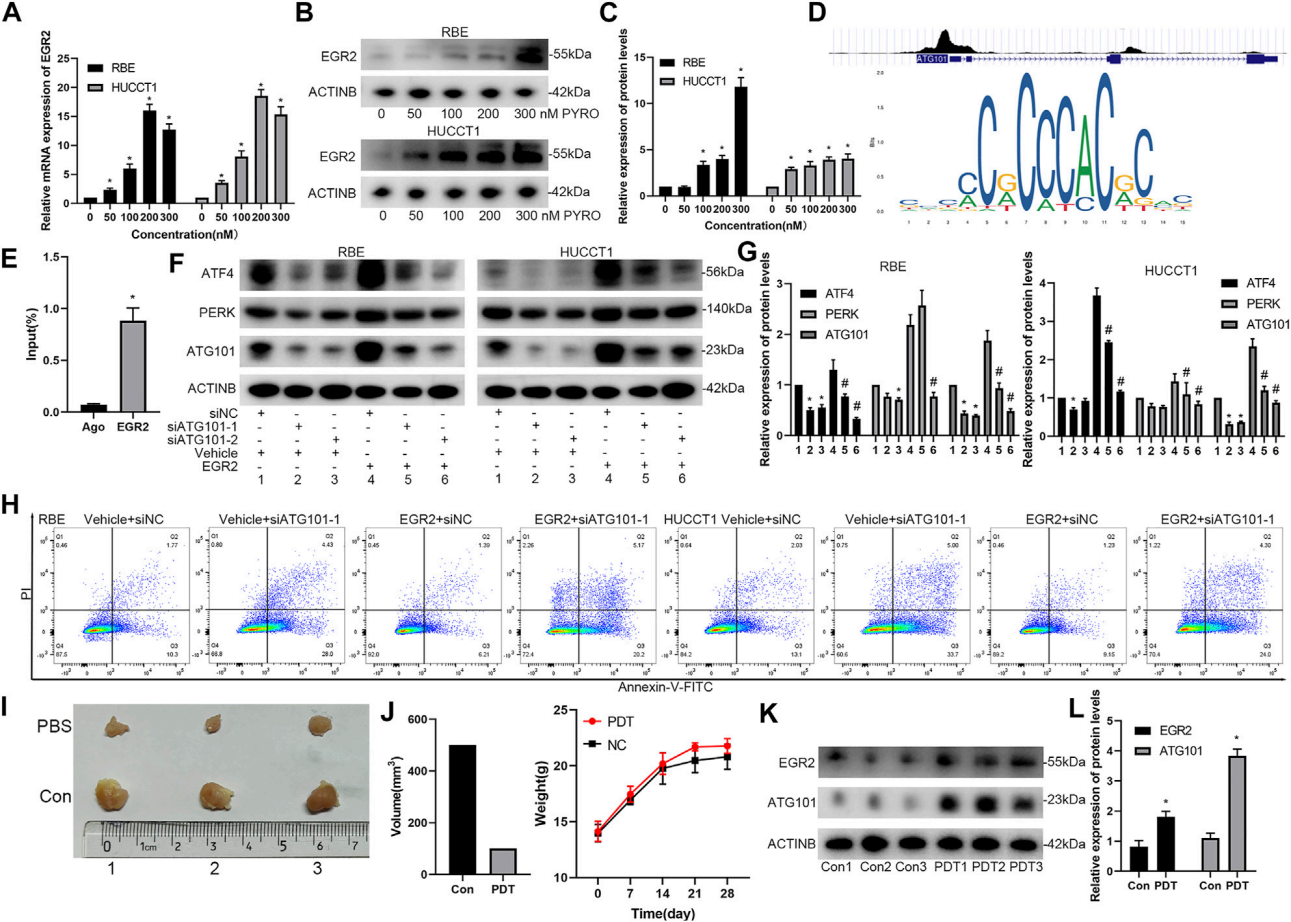
EGR2 regulates the expression of ATG101 and apoptosis rate after PYRO-PDT treatment. **(A)** Through the Cistrome tool in UCSC genome browser, the binding peak of EGR2 and ATG101 promoter were shown in (i); a sequence matrix of EGR2 binding to AT101 promoter was shown in JASPAR database (ii). **(B,C)** After processing RBE and HUCCT1 cell lines in 6-well plates with different concentrations of PYRO-PDT for 4 h, the mRNA and protein levels of EGR2 were determined by qRT-PCT or Western blot. **(D)** is a histogram of the relative expression of proteins in each group in **(C)**. **(E)** The RBE and HUCCT1 cell lines overexpressing EGR2 were constructed by lentivirus, and then transfected by siNC, siATG101-1, siATG101-2, respectively. The expression of ATF4, PERK and ATG101 were detected by western blot after 48 h of transfection. **(F)** is a histogram of the expression of proteins in each group in **(E)**. * Compared with group 1; ^#^ compared with group 4. **(G)** ChIP-qPCR assays were performed to find the EGR2 required for binding of the ATG101 promoter. Ago: Agarose group; EGR2: Anti-EGR2 group. **(H)** The apoptosis rate in different levels of EGR2 and ATG101 in RBE and HUCCT1 cell lines was assessed by Annexin-V-FITC/PI staining and flow cytometry. **(I)** Cholangiocarcinoma subcutaneously xenograft model was used to evaluate the effect of PYRO-PDT group and PBS (Con) control group on tumors. RBE cells (2.5*107/ml, 200 ul/each) were injected into the left groin of nude mice. After 12 days, we calculated the tumor size every 3 days. When the tumor size reached 1 cm^3^, the mice were sacrificed, and the tumors were removed for further detection. **(J)** is the statistical graphs of tumor volume and body weight in PDT and Con groups, respectively. **(K)** After the tumor was taken out, western blot was used to detect the effect of PDT treatment on the changes of EGR2 and ATG101. **(L)** is a histogram of the expression of proteins in each group in **(K)**. *, ^#^ *p* < 0.05.

On the premise of determining the relationship between ATG101 and PDT, we further explored the relationship between ATG101-related transcription factors and PDT. EGR2 is a transcription factor that is often highly expressed during early cell stress. We determined that the mRNA and protein of EGR2 were up-regulated in a concentration-dependent manner after PYRO-PDT treated RBE and HUCCT1 cells (Figures 10A–C). According to the transcription factors enriched by GSEA in Figure 7 and the results of PDT treatment, we speculate that EGR2 may have a regulatory relationship with ATG101. The cistrome tool of the UCSC genome browser found that EGR2 was significantly enriched in the promoter region of ATG101, and the sequence of this region was imported into the JASPAR database to further support the possibility of EGR2 collection ATG101 promoter (Figure 10D). ChIP-PCR further confirmed the binding of EGR2 to the ATG101 promoter (Figure 10E). In the constructed RBE and HUCCT1 cells that stably overexpress EGR2, EGR2 can up-regulate the expression of ATG101, ATF4 and PERK, and inhibiting ATG101 can reverse the indirect regulation of ATF4 and PERK by EGR2, which suggests that EGR2 acts as an upstream regulator of ATG101 and ATF4 (Figures 10F,G). In the same group, the apoptosis experiment also confirmed the resistance of EGR2 overexpression to apoptosis. On the contrary, downregulation of ATG101 can increase apoptosis (Figure 10H). We performed PYRO-PDT in subcutaneously xenograft model to confirm the effect of PDT and the changes of ATG101 and EGR2. The results showed that the size of subcutaneous tumors in the PDT group was lower than that in the Con group (PBS treatment) (Figures 10I,J). In addition, the protein levels of ATG101 and EGR2 in the tumors of the PDT group were also higher than those of the Con group (Figure 10L). Combining the previous results, we believe that ATG101 and EGR2 are PDT resistance factors.

## Discussion

ATG101, a novel mammalian autophagy factor thought to directly interact with Atg13, was first identified by Hosokawa in 2009 (Hosokawa et al., 2009). Previous studies regarding ATG101 were mainly in the field of autophagy. ATG101 is an essential gene for the initiation of autophagy and might be a potential therapeutic target in diseases involving endothelial injury (Du et al., 2017). Meanwhile, ATG101 also functions together with FIP200 ATG13 and ULK (Hosokawa et al., 2009). Regulating ULK1 to regulate autophagy/autophagy-related cell death (ACD) may be effective in the treatment of triple-negative breast cancer (Ouyang et al., 2017). Moreover, ATG101 is essential for tissue homeostasis in both adult brains and midguts (Guo et al., 2019) and mediates GSN and OAS2, which are positively and negatively associated with the recurrence of colorectal cancer, respectively (Kim et al., 2018). Some scholars have used 7 ATG-related genes to establish the prognostic risk characteristics of hepatocellular carcinoma (Mao et al., 2020). Another previous study showed that stable knockdown of ATG5 can significantly inhibit the occurrence and progression of colorectal cancer tumours *in vivo* (Qureshi-Baig et al., 2020). In addition, ATG genes are mainly associated with the regulatory mechanism of cell-specific gene expression in CHOL (Kagaya et al., 2001). However, frameshift mutations of ATG-related genes may affect tumour progression by regulating autophagy in gastric cancer and colorectal cancer (Kang et al., 2009). These studies are consistent with the results of our study. Therefore, ATG101 is expected to be a diagnostic and prognostic marker of different tumours. However, there are no studies on the significance of ATG101 in pan-cancer or its role in the immune microenvironment. In this study, we explored for the first time the relationship between ATG101 and different tumours from a pan-cancer perspective.

Cancer neoantigens are generally considered to be a new class of antigens produced as a result of individual cancer cell mutations. Because of their immunogenicity and low expression in normal tissues, cancer neoantigens are considered to be an important target for cancer immunotherapy (Li et al., 2017). Changes in the abundance and mutation profile of ATG101 in various cancers leads to the emergence of new antigenic epitopes. Some initial, authoritative clinical trials revealed that dendritic cell-related neoantigen vaccines are safe and can induce CD8^+^ and CD4^+^ neoantigen-specific T cell responses, which have broad application prospects (Peng et al., 2019). In fact, dendritic cells (DCs) are an important part of the immune microenvironment. An increasing number of studies have revealed that DC vaccines with specific genes (such as ATG101) can effectively initiate adaptive cytolytic immune responses. For example, one prospective study confirmed the safety and effectiveness of vaccines against cancer neoantigens. The cancer neoantigen vaccine can induce specific CD4^+^ and CD8^+^ T cells to target specific patient tumours, and in further clinical trials, it is expected that the effect of the vaccine will be satisfactory (Ott et al., 2017). In addition, Beatriz confirmed in patients with advanced melanoma that the DC vaccine can improve naturally occurring neoantigen-specific immunity and discovered a new human leukocyte antigen (HLA) neoantigen (Carreno et al., 2015). In addition, there is an urgent need to develop vaccines that target DCs or use them to present antitumour antigens in clinical practice (Harari et al., 2020).

In this study, we attempted to deeply explore the relationship between immunity and ATG101 expression; therefore, we analysed the correlation between six types of immune cells, B cells, CD4^+^ T cells, CD8^+^ T cells, neutrophils, macrophages and dendritic cells, and the expression levels of ATG101. We found that in LIHC, LUSC and PCPG, the expression of ATG101 was positively correlated with all six immune cells. ATG101 may be a potential target for immunotherapy of these cancers. We further studied the relationship between ATG101 expression and neoantigens in various cancers. Our results showed that ATG101 expression was positively correlated with neoantigens in BRCA, KIRC, READ, HNSC, LIHC, SKCM and CESC. LIHC (liver hepatocellular carcinoma) was the only cancer in which there was a positive correlation among all six immune cells and neoantigens. The LHIC tumour microenvironment is complex and changeable. The TME affects the process of cancer cell antigen presentation by expressing tumour antigens, thereby allowing tumour cells to evade effective tumour treatment methods (He et al., 2020). The complex tumour immune microenvironment of the LHIC is also one of the main causes of the heterogeneity of the treatment response to immune checkpoint blockers such as PD-1 and CTLA-4 blockade in LHIC patients with the same TNM stage (Prieto et al., 2015). We also studied the relationship between ATG101 expression and immune checkpoint genes. In BRCA, COAD, LIHC, LUAD and PCPG, we found that ATG101 and other immune checkpoint genes were significantly coexpressed. However, the expression of ATG101 was negatively correlated with all forty-seven immune checkpoint genes in CHOL, DCBC, TGCT and UCS. Therefore, ATG101 may be a potential biomarker to determine the prognosis of LHIC patients.

TMB is the total number of mutations per megabase in cancer, representing tumour mutation quantity. The more tumour mutations there are, the greater the differences between the cancer cells and normal cells. That is, the higher the value of TMB is, the more likely the cancer is to be recognized and attacked by the immune system and the better the response to immunotherapy. In recent years, TMB has been considered a biomarker for immunotherapy and an important evaluation index directly related to the efficacy of immune checkpoint therapy, such as PD-1 and CTLA-4 blockade (Jardim et al., 2021). Similarly, the formation of MSI caused by DNA MMR protein defects results in the accumulation of mutations and the production of neoantigens. MSI is also an important marker that determines the effectiveness of immunotherapy (Petrelli et al., 2020). Our results showed that ATG101 expression was significantly correlated with both MSI and TMB only in COAD, HNSC and SKCM. This indicates that there is a significant negative correlation between ATG101 expression and MSI and TMB in these cancers. Our results are consistent with the results of previous studies. Maria demonstrated that immunogenic ileal apoptosis contributes to the prognosis of chemotherapy-treated COAD (Roberti et al., 2020). In addition, Simone’s research showed that CD8^+^ T cell-related therapies can be used for anticancer immunotherapy for SKCM (Park et al., 2019). Similar results have also been verified in HNSC (Oliva et al., 2019).

In recent years, an increasing number of studies have shown that DNA methylation is highly related to the diagnosis, prognosis and prediction of the response to therapies for tumours, such as NSCL, colorectal cancer, hepatocellular carcinoma and metastatic breast cancer (Győrffy et al., 2016; Tse et al., 2017; Mahmood and Rabbani, 2019). DNA methylation is one of the most common epigenetic events in the mammalian genome. Normal DNA methylation can maintain the normal functions of the body, such as the stability of the genome structure, normal embryonic development and cell differentiation, while abnormal DNA methylation will lead to tumorigenesis (Koch et al., 2018). Some studies have indicated that the ATG gene may be involved in the development and progression of various diseases through promoting demethylation, regulating a variety of cellular functions and signalling pathways (Fu et al., 2018; Chen et al., 2019a; González-Rodríguez et al., 2020). These studies indicated that DNA methylation may play a critical role in ATG gene expression. This study also revealed that the expression of several DNA methylation/demethylation factors has a significant correlation with ATG101 methylation status.

In this study, we found that the correlation between the expression levels of DNA methyltransferase and ATG101 varied in different tumours. Moreover, we explored the function of ATG101 in COAD and LIHC. Twenty-one common genes related to ATG101, COAD and LIHC were obtained. Our results are in agreement with previous studies. It was concluded that targeting STAT5-and STAT6-related signalling pathways can inhibit the proliferation of CRC cells and induce CRC cell apoptosis (Jiang et al., 2019), and Dong suggested that STAT5A, STAT5B and STAT6 expression may be potential prognostic markers of hepatocellular carcinoma (Dong et al., 2019). MiR-532-3p can inhibit the progression of colorectal cancer by regulating ETS1-related signalling pathways (Gu et al., 2019). Meanwhile, WTAP plays an important role in the progression of hepatocellular carcinoma by affecting the epigenetic modifications of ETS1 (Chen et al., 2019b). Chen concluded that MJD1C can affect colorectal cancer metastasis by targeting ATF2 (Chen et al., 2018). However, further research is still needed to explore ATG101 epigenetic changes and its potential functions, which may contribute to the development of new cancer treatments and the discovery of new markers to predict the prognosis of patients with cancer.

## Data Availability

The datasets presented in this study can be found in online repositories. The names of the repository/repositories and accession number(s) can be found in the article/Supplementary Material.

## Glossary

ACC: Adrenocortical carcinoma
BLCA: Bladder Urothelial Carcinoma
BRCA: Breast invasive carcinoma
CCLE: Cancer Cell Line Encyclopedia
CESC: Cervical squamous cell carcinoma and endocervical adenocarcinoma
CHOL: Cholangiocarcinoma
COAD: Colon adenocarcinoma
DFS: disease free survival
DLBC: Lymphoid Neoplasm Diffuse Large B-cell Lymphoma
DSS: disease special survival
ESCA: Esophageal carcinoma
FPPP: FFPE Pilot Phase II FFPE
GBM: Glioblastoma multiforme
GBMLGG: Glioma
GEO: Gene Expression Omnibus
HNSC: Head and Neck squamous cell carcinoma
KICH: Kidney Chromophobe
KIPAN: Pan-kidney cohort (KICH + KIRC + KIRP)
KIRC: Kidney renal clear cell carcinoma
KIRP: Kidney renal papillary cell carcinoma
LAML: Acute Myeloid Leukemia
LGG: Brain Lower Grade Glioma
LIHC: Liver hepatocellular carcinoma
LUAD: Lung adenocarcinoma
LUSC: Lung squamous cell carcinoma
MESO: Mesothelioma
MSI: microsatellite instability
OS: Overall survival
OV: Ovarian serous cystadenocarcinoma
PAAD: Pancreatic adenocarcinoma
PCPG: Pheochromocytoma and Paraganglioma
PFS: progression free survival
PRAD: Prostate adenocarcinoma
READ: Rectum adenocarcinoma: Esophageal carcinoma
READ: Rectum adenocarcinoma
SARC: Sarcoma
SKCM: Skin Cutaneous Melanoma
STAD: Stomach adenocarcinoma
STES: Stomach and Esophageal carcinoma
TCGA: The Cancer Genome Atlas
TGCT: Testicular Germ Cell Tumors
THCA: Thyroid carcinoma
THYM: Thymoma
TMB: tumor mutation burden
UCEC: Uterine Corpus Endometrial Carcinoma
UCS: Uterine Carcinosarcoma
UVM: Uveal Melanoma
