# Supplementary material for: Comprehensive Analysis of the Potential Immune-Related Biomarker ATG101 that Regulates Apoptosis of Cholangiocarcinoma Cells After Photodynamic Therapy
Source: Front Pharmacol. 2022 May 3;13:857774. doi: 10.3389/fphar.2022.857774 (PMC9110647; doi:10.3389/fphar.2022.857774)
Supplement: Supplementary file 1 [file DataSheet1.docx]

**Supplementary materials**

**Table S1. PCR-primer information**

| **No.** | **Gene** | **Sequence** |
| --- | --- | --- |
| 1 | ATG101-F | AAGTTCCACTACAAGAAGGAGG |
| 2 | ATG101-R | GATCCAGTTCCTCAGAAGAGAC |
| 3 | EGR2-F | CACAGCCTCATCCAGCGTCAC |
| 4 | EGR2-R | GAGAGTACAGGTGGTCCAGGTCAG |
| 5 | ATF4-F | ATGGATTTGAAGGAGTTCGACT |
| 6 | ATF4-R | AGAGATCACAAGTGTCATCCAA |
| 7 | PERK-F | ATAGCCCTCACCATTTGC |
| 8 | PERK-R | GGGGACTTTCCTTCTTCTG |
| 9 | GAPDH-F | GTATCGTGGAAGGACTCATGAC |
| 10 | GAPDH-R | ACCACCTTCTTGATGTCATCAT |

**Figure S1.** The relationship between the expression level of ATG101 and the immune score of the algorithm in cancer patients


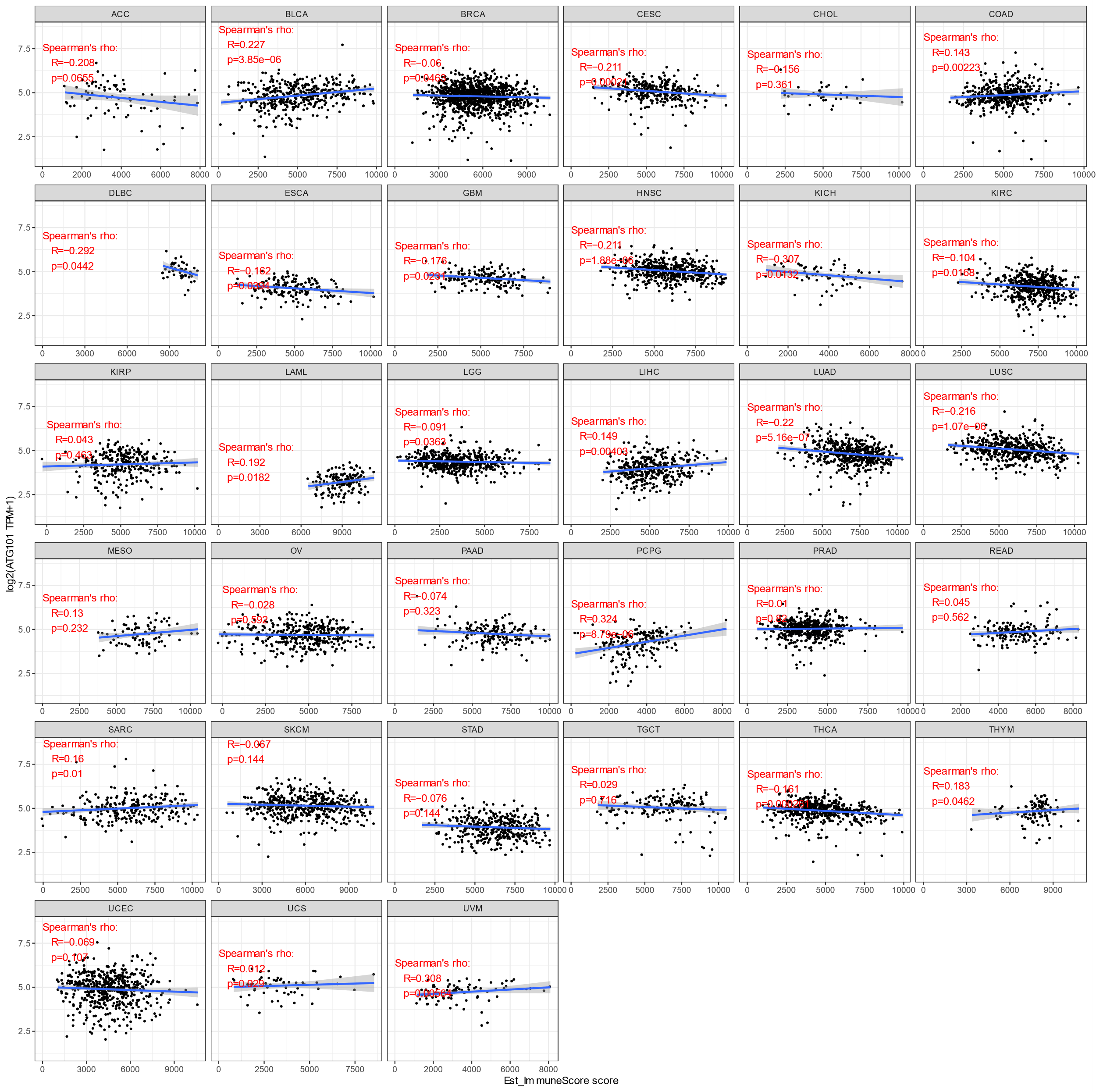


**Figure S2.** The relationship between the expression level of ATG101 and the stromal score of the algorithm in cancer patients


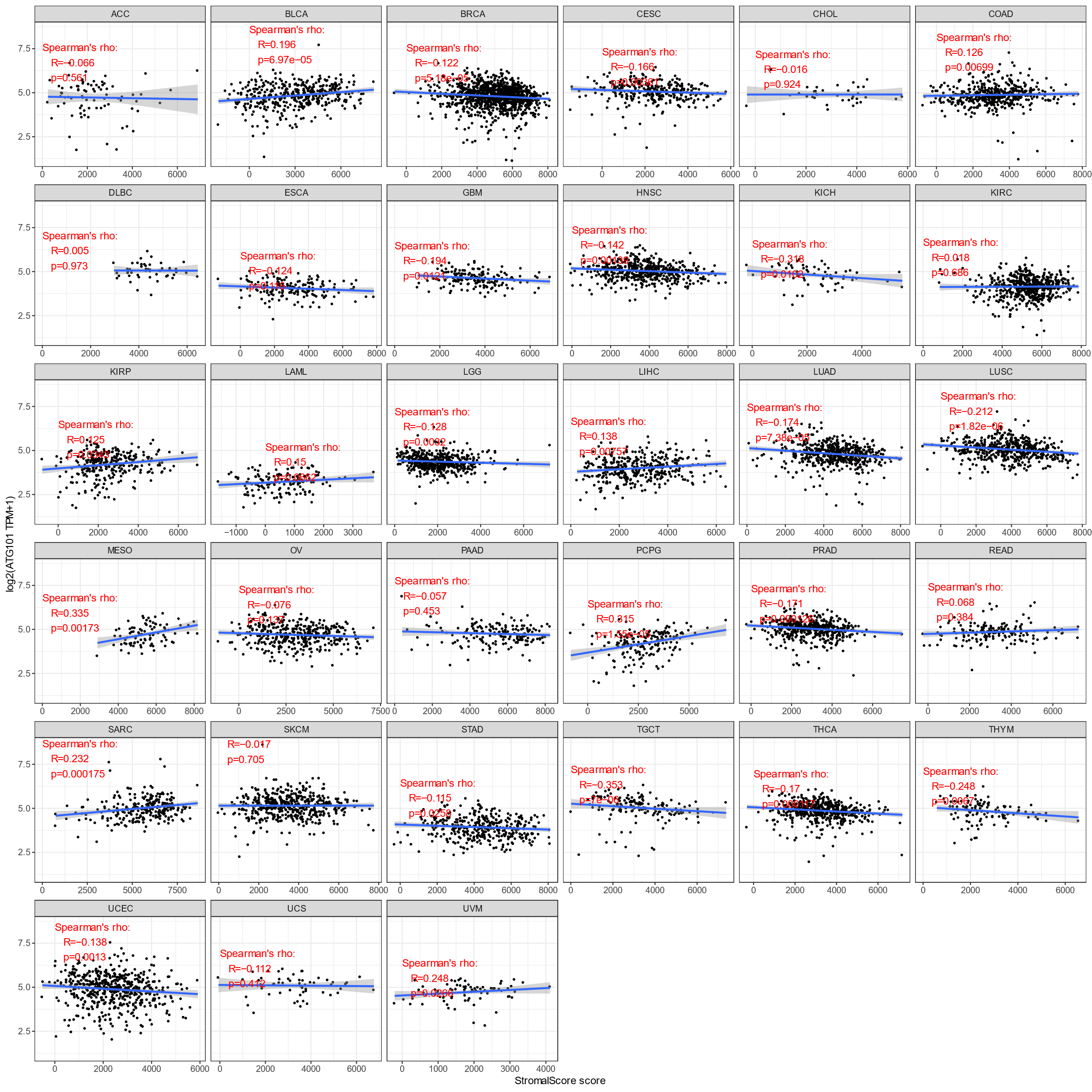


**Figure S3.** The relationship between the expression level of ATG101 and the ESTIMATE score of the algorithm in cancer patients


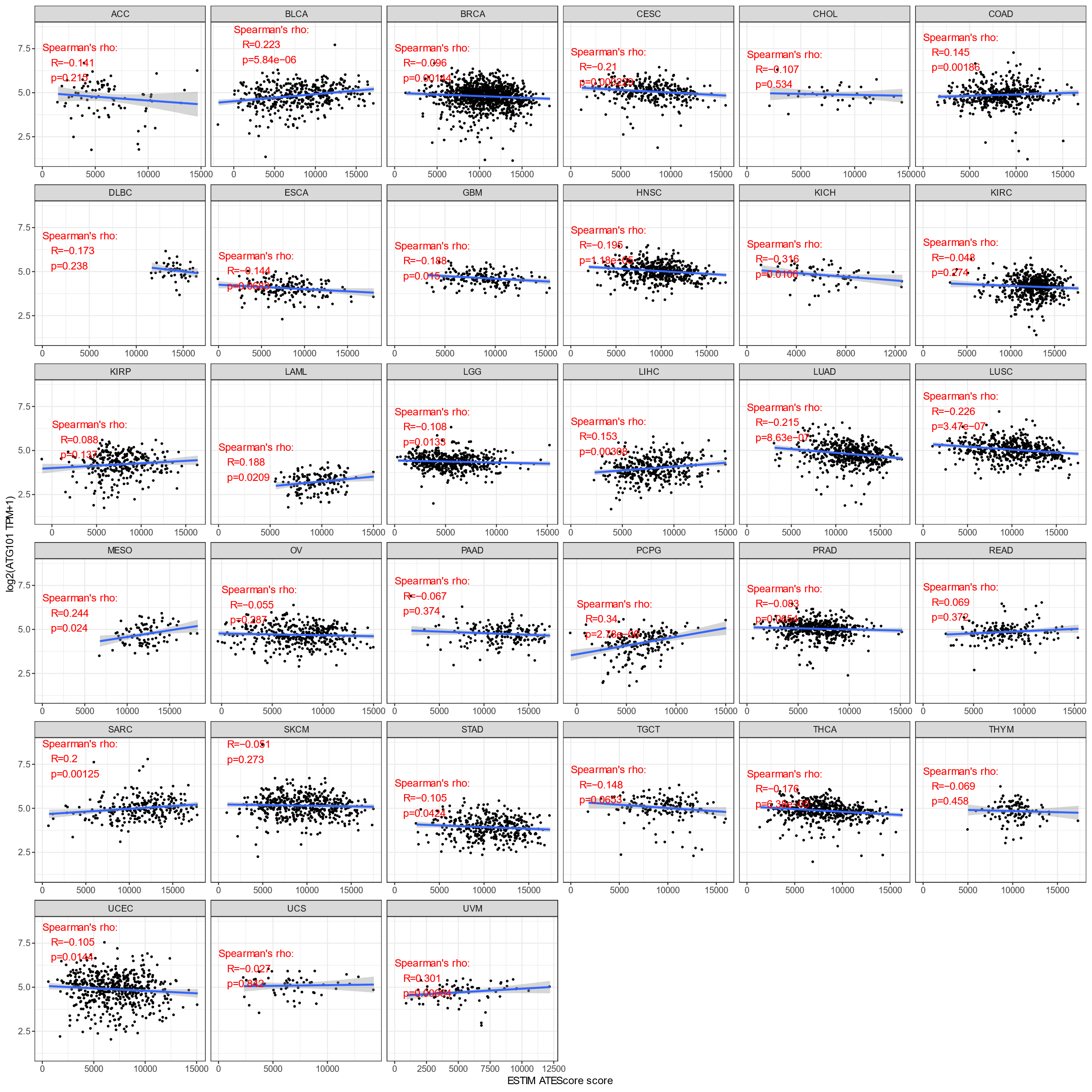


**Figure S4.** Correlation between tumour neoantigen and ATG101 expression level.


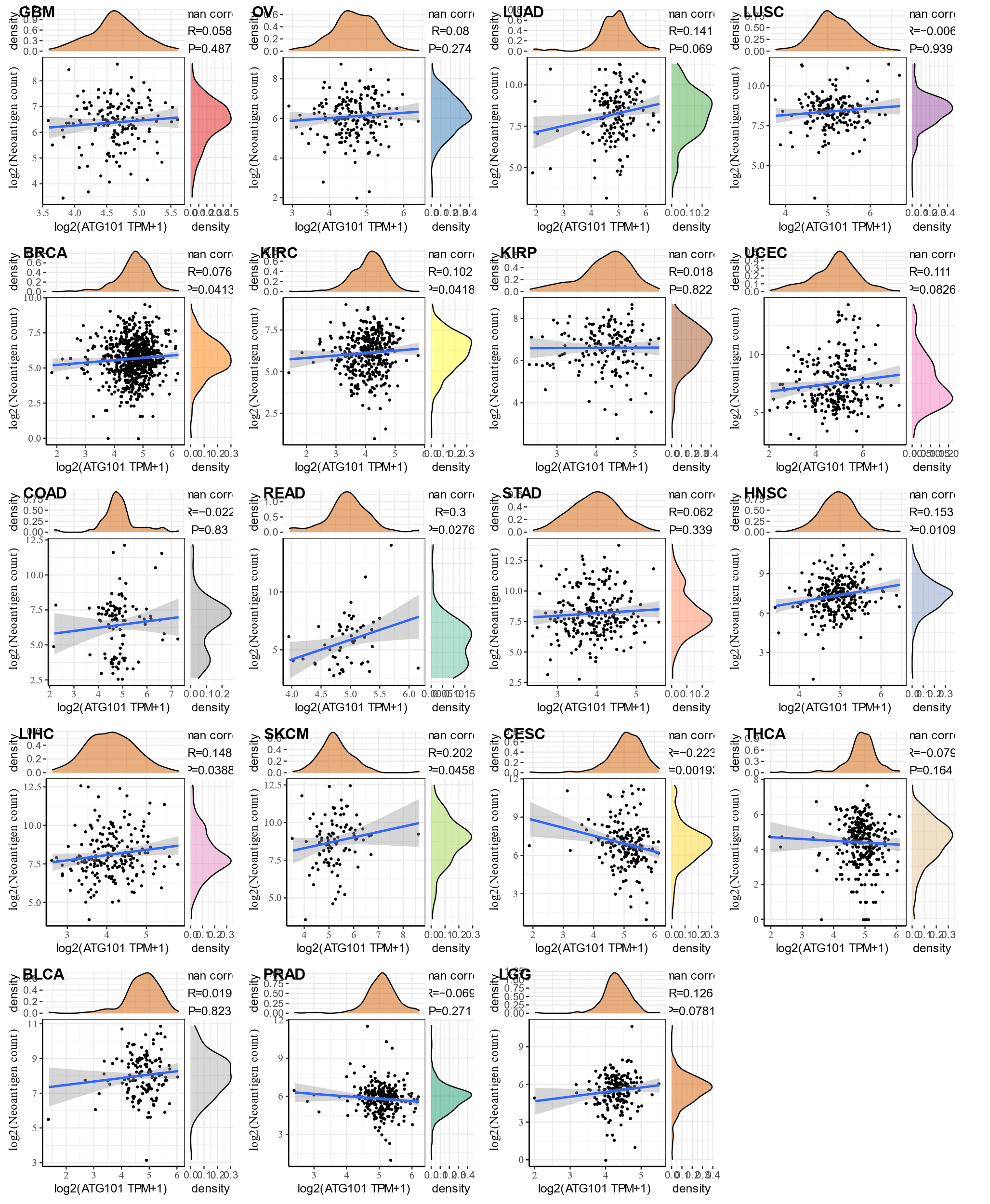


**Figure S5.** The correlation between ATG101 and ATF4 or PERK (data derived from TCGA-CHOL in GEPIA database).

**
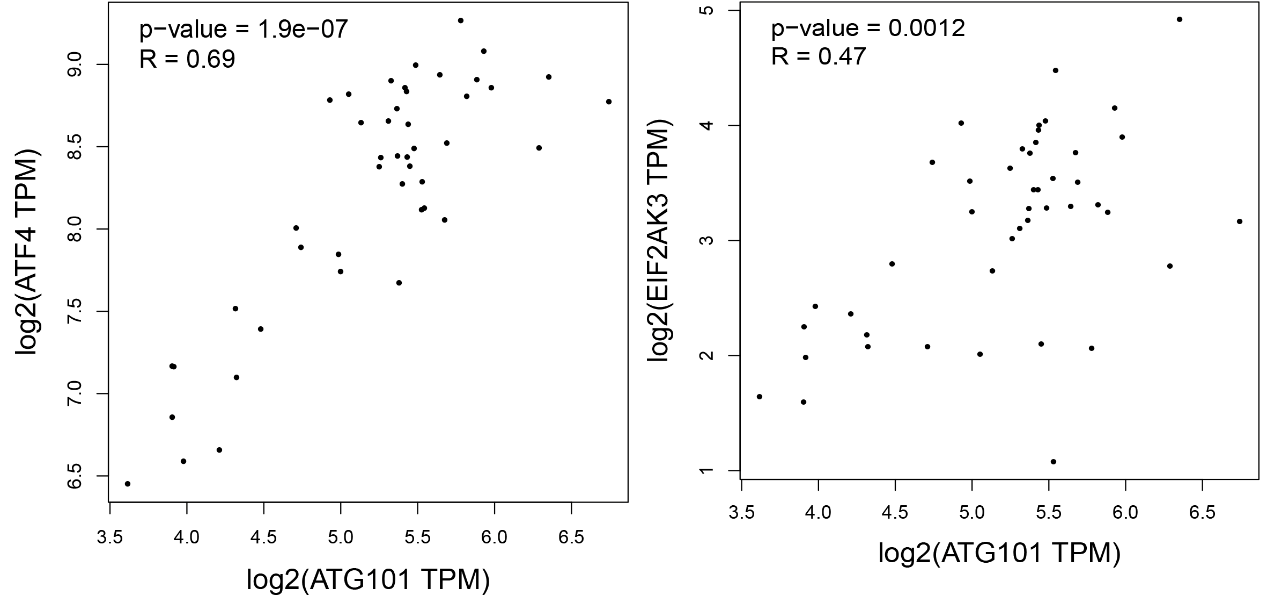
**
